# Development and Validation of PubMed and Ovid MEDLINE Search Filters for Exposure Pathways Linking Climate Change With Public Health

**DOI:** 10.1002/cesm.70105

**Published:** 2026-08-31

**Authors:** Maria‐Inti Metzendorf, Ina Monsef, Katherine Jones, L Susan Wieland, Heidrun Janka, Camila Escobar Liquitay, Denise Thomson

**Affiliations:** ^1^ Cochrane Planetary Health Thematic Group, Institute of General Practice, Medical Faculty of the Heinrich‐Heine‐University Düsseldorf Düsseldorf Germany; ^2^ Institute of Public Health, Faculty of Medicine and University Hospital Cologne University of Cologne Cologne Germany; ^3^ School of Health and Related Research (ScHARR) University of Sheffield Sheffield UK; ^4^ Cochrane Complementary Medicine University of Maryland School of Medicine Baltimore Maryland USA; ^5^ Institute for Complementary and Integrative Medicine University Hospital Zürich and University of Zürich Zürich Switzerland; ^6^ Department of Research Instituto Universitario Hospital Italiano de Buenos Aires Buenos Aires Argentina; ^7^ Department of Pediatrics, Cochrane Planetary Health Thematic Group University of Alberta Edmonton Canada

**Keywords:** climate change, evidence synthesis, information storage and retrieval, MEDLINE, public health, search filters, systematic reviews

## Abstract

**Introduction:**

Climate change has major health impacts to which researchers and policymakers need to respond. The volume and multidisciplinary nature of climate‐health evidence pose challenges to its comprehensive identification. Search filters are currently not available for this topic. Our aim was to empirically develop sensitivity‐maximizing climate‐health search filters for two interfaces of the MEDLINE database.

**Methods:**

Climate change impacts health via several exposure pathways involving distinct mechanisms: extreme weather events, heat stress, air quality, water quality and quantity, food supply and safety, vector distribution and ecology, and social factors. Using the relative recall method, we established a gold standard by extracting included studies of 110 evidence syntheses and classifying them into exposure pathways. From this set, we empirically derived and validated filters per pathway.

**Results:**

Based on 1572 primary studies indexed in PubMed, we developed filters with a sensitivity of 95%, 97% and 99% for six of the seven major climate‐health exposure pathways.

**Conclusion:**

We designed the first empirically derived search filters for climate‐health pathways, enabling sensitive retrieval despite acknowledged limitations. Our filters can be applied to different types of research questions at the intersection of climate and health.

## Key Messages

- The identification of evidence linking climate change with health in literature databases such as MEDLINE presents challenges and search filters are lacking for this topic.
- We present search filters with graded sensitivities for six of seven major climate‐health exposure pathways.
- The development of a filter for the pathway ‘social factors’ was not viable, suggesting that it requires other databases and complementary search methods.
- The search filters will help health information professionals and researchers to comprehensively identify relevant studies with a relationship to climate change.
- The filters can be applied to different types of research questions (interventions, associations, impacts, diseases, populations, regions) as they focus on major exposure pathways.

## 1 Introduction

Climate change is considered the greatest public health challenge of the 21st century [1, 2]. It causes long‐term shifts in global and regional climate patterns, primarily characterised by alterations in temperature, precipitation, and other climatic factors attributed to the anthropogenic influence on greenhouse gas (GHG) emissions, with profound impacts on ecosystems, human societies, and economies worldwide [1, 3]. The climate crisis results in rising temperatures and sea levels, and increased intensity, frequency, and duration of extreme weather events, which impact human health through various exposure pathways. Extreme weather events, heat stress, air quality, water quality and quantity, food supply and safety, vector distribution and ecology, and social factors are associated with multiple health outcomes such as injuries, heat stress, exacerbation of respiratory illnesses, increase of water‐, food‐ and vector‐borne diseases, adverse mental health impacts, forced migration and death [3, 4].

Policymakers around the world must not only work within health systems on the regional and country level, but also with other sectors of governance, such as municipalities, local communities and organisations, disaster response and natural resource authorities, as well as agricultural departments to help protect populations systems [5]. To do this effectively they must develop evidence‐informed responses to both reduce fossil fuels and GHG emissions and address the wide range of impacts that climate change is having on all aspects of life on this planet, including public health and health systems [5, 6].

High‐quality evidence syntheses are a vital resource for policy‐making, as a consolidated guide through a vast amount of pertinent policy‐relevant research. They can range in scale from the global reports produced by the Intergovernmental Panel on Climate Change [7] and the Lancet Countdown [1] to more focused publications such as systematic reviews or evidence gap maps [8]. Evidence syntheses can be used to collate information about the benefits, adverse effects, and costs of interventions, as well as identifying knowledge gaps and research priorities [9].

The quality of evidence syntheses rests in part on the comprehensiveness of the included evidence, which in turn depends on the quality of the search strategies used to identify the evidence [10]. Developing search strategies for health impacts of climate change can be complicated by the volume and multidisciplinary nature of the evidence on the one hand, and by the inconsistent attribution of relevant studies to climate change on the other. The latter is one of the key challenges encountered during identification of studies linking climate change and health, as relevant studies do not consistently mention climate change as a causal or aggravating factor of specific health‐related exposures. For example, water quality deterioration due to floods that occur more frequently and intensely or a change in vector distribution and ecology because of rising temperatures.

In addition, the literature on health‐related aspects of climate change covers several distinct fields: 1. impacts (effects on human health through several exposure pathways), 2. mitigation (interventions to reduce both fossil fuels and GHG emissions, as well as the effects of these measures, including their health co‐benefits), and 3. adaptation (interventions to adapt to the impacts of climate change and reduce its detrimental effects on health, including vulnerability assessments and preventative approaches) [1, 7].

The increasing number of evidence syntheses being published on the health‐related links to climate change suggests a need for improved methods for their conduct [11]. Search filters—predefined and ideally empirically derived and validated combinations of search terms on a specific subject or methodological design (e.g. randomised controlled trials)—play an important role in ensuring robust search methods [12]. They are used by information specialists and librarians who support different research endeavours and specifically the search process for evidence syntheses. The InterTASC ISSG search filters resource (https://sites.google.com/a/york.ac.uk/issg-search-filters-resource/home) did not include climate change‐related filters up to April 2026. As each exposure pathway linking climate change and health involves distinct terminology and mechanisms, the empirical development of pathway‐specific filters promises more comprehensive retrieval than designing conceptual search strategies.

Therefore, the objective of this methodological study is the development and validation of sensitivity‐maximizing subject search filters for established exposure pathways through which climate change impacts human health for the MEDLINE database with two main search interfaces (PubMed and Ovid). By building filters for the pathways linking climate change and health, we aim to enable health information professionals and researchers to 1. deploy them for a diverse range of topics, which can comprise the assessments of impacts, the identification of preventative measures and vulnerable populations, supporting adaptation and resilience‐building efforts, as well as policy prioritisation and 2. overcome inherent challenges encountered during identification of relevant climate‐health related studies due to the inconsistently mentioned link to climate change.

## 2 Materials & Methods

To develop search filters for exposure pathways through which health is influenced by climate change, we focused on seven established pathways linking climate change and health: air quality, extreme weather events, food supply and safety, heat stress, social factors, vector distribution and ecology, and water quality and quantity [3, 6]. Definitions of the pathways are presented in Table 1.

**Table 1:** Exposure pathways through which health is influenced by climate change (CC).

Exposure pathway	Definition (Salas et al.[3])	Examples (Haines/Ebi[6])
1) Air quality	Air quality deteriorates with climate change (CC) through many pathways; hotter temperatures result in higher levels of ground‐level ozone, wildfires result in increased levels of particulate and other air pollutants, and plants produce more pollen and allergens as a result of higher CO_2_ concentrations in the atmosphere [†]. In addition to releasing CO_2_, the burning of fossil fuels releases particulate air pollutants.	* Exacerbations of asthma and other respiratory diseases * Respiratory allergies * Cardiovascular disease * Bacterial meningitis and invasive bacterial disease (linked to airborne dust)
2) Extreme weather events (floods, droughts, storms)	Extreme weather, including increasingly intense storms leading to coastal flooding with rising sea levels, can result in a wide range of health effects. These may include death or injuries, worsening of existing medical illnesses, spread of waterborne diseases, and adverse effects on mental health. Extreme events can also lead to wide‐ranging negative consequences for health care delivery.	* Injuries * Fatalities * Mental health effects
3) Food supply and safety	As atmospheric levels of CO_2_ increase, the nutritional value (protein and essential minerals) of vital food crops decreases. Warmer temperatures and more extreme weather can also impair food safety (as climate‐sensitive pathogens cause foodborne illness and chemical contamination) and distribution (e.g. by disrupting transportation).	* Undernutrition * Salmonella food poisoning and other foodborne diseases * Mycotoxin effects * Diarrhoea/E. coli * Diarrhoea/V. cholerae
4) Heat stress	As the planet warms, heat waves are becoming more frequent and lasting longer—placing people at increased risk for heat‐related illnesses such as heat stroke. Heat‐related illness can be seen at temperatures much lower than those characteristic of a heat wave.	* Heat‐related illness and death * Cold spell, cold effects, extreme cold weather
5) Social factors	Adverse effects of CC—including effects on availability of water and food, extreme weather, and coastal flooding—are causing forced displacement of populations within their own countries (internally displaced persons) or across national borders (climate refugees) and leading to conflict and violence.	* Physical and mental health effects of violent conflict and forced migration (complex and context‐specific risks)
6) Vector distribution and ecology	CC is altering the geographic distributions and life cycles of insect vectors [‡], such as mosquitoes and ticks, with associated increases in the numbers and range of vector borne diseases and the emergence of new diseases. These changes have important implications for several diseases such as Lyme disease, West Nile virus, malaria, and dengue.	* Chikungunya (M) * Dengue (M) * Encephalitis (various forms) * Hantavirus infection (R) * Lyme disease (T) * Malaria (M) * Rift Valley fever (M) * West Nile virus infection (M) * Zika virus infection (M) * Ross River fever (M) * Crimean‐Congo haemorrhagic fever (T) * Leishmaniasis (S)
7) Water quality and quantity	CC may compromise water quality through several pathways, such as favouring the growth of organisms that cause waterborne gastrointestinal illness and boosting the growth of harmful algae, Cyanobacteria, Escherichia coli and other bacterial strains, and viruses. Clean water may not even be available owing to drought or contamination after extreme weather and storm surges.	* Campylobacter infection * Cholera * Cryptosporidiosis * Harmful algal blooms * Leptospirosis * Norovirus infection * Typhoid fever * Rotavirus infection * Diarrhoea/e. coli * Melioidosis * Hepatitis E * Dysentery

*Note:* Column 2 according to [3] and column 3 according to [6]. Additions in italics by the authors, based on the studies of our reference set adjudicating this health outcome to the pathway.

Abbreviations: M, mosquitoes; R, rodents; S, sandflies; T, ticks.

^†^ Increased pollen concentrations in the air also result from stronger and faster spread of certain heat‐ and drought‐resistant plant species due to CC (e.g. ragweed, Ambrosia artemisiifolia).

^‡^ Animal disease vectors also comprise other taxonomic groups than insects, e.g. ticks (mites, arachnids), other arthropods, and also mammals (e.g. bats, rats and mice).

### 2.1 Building the Gold Standard

**Figure 1:**
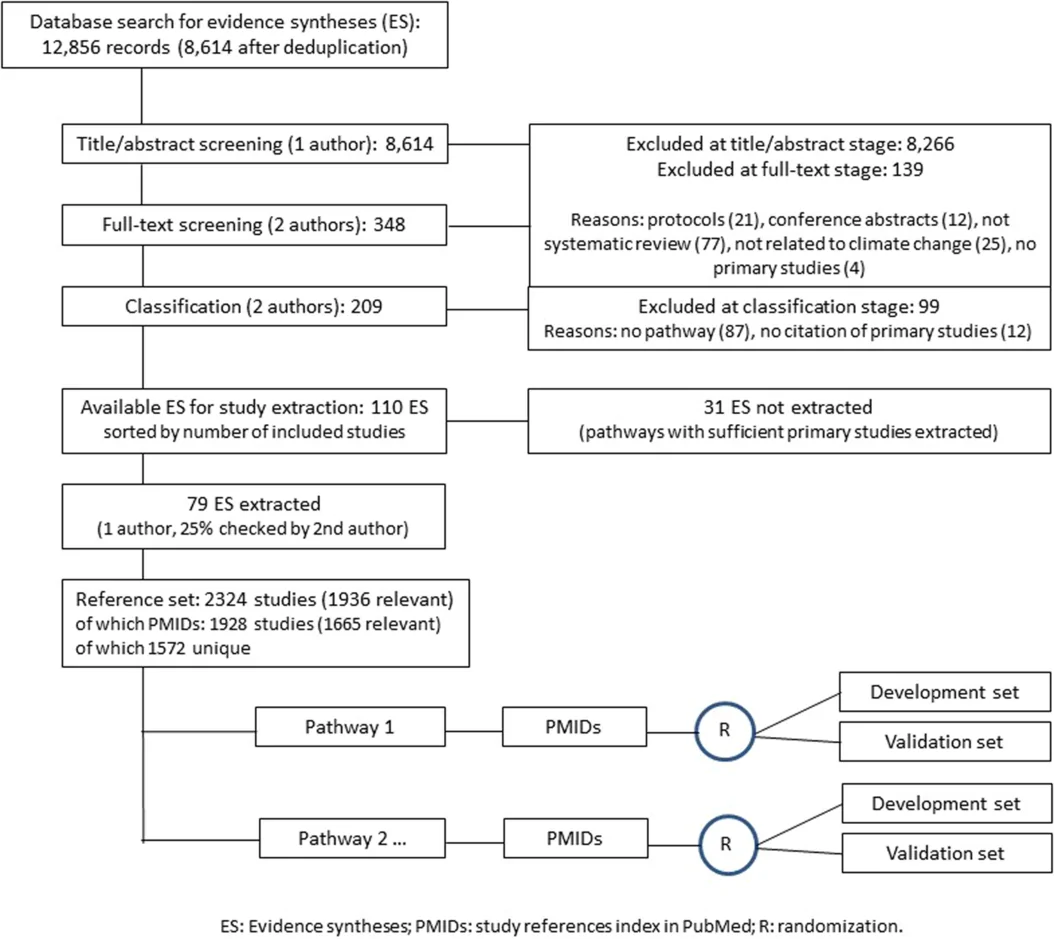
Methodological approach to establish the reference, development, and validation sets. ES, Evidence syntheses; PMIDs, study references index in PubMed; R, randomization.

To empirically derive search filters from the literature, we used the relative recall method [13] to establish a representative set of primary studies included in existing evidence syntheses on the health impacts of climate change. It implies the construction of a “gold standard” (in the following termed ‘reference set’) of relevant studies on the topic, which is divided into two groups. One group of references, called “development set,” is used to develop a filter by analyzing the text words (title/abstract) and the controlled vocabulary (‘Medical Subject Headings’ assigned to references in the MEDLINE database), and the second group of references, called “validation set,” is used to test the performance of the developed filter in retrieving those references [14, 15]. Our methodological approach to establish the reference, development, and validation sets for the search filters is depicted in Figure 1.

#### 2.1.1 Search for Evidence Syntheses

To identify potentially relevant evidence syntheses, we used a sensitive search strategy developed and peer reviewed by experienced information professionals (DKL, DS) independent from the author team. This strategy was run in MEDLINE, Embase, CINAHL, the Cochrane Database of Systematic Reviews and ProQuest Dissertations and Theses on February 10, 2021 and limited to English language evidence syntheses published from 1999 onwards (see Appendix 1). The search yielded 12,856 records, resulting in 8614 records after deduplication in EndNote.

#### 2.1.2 Inclusion and Classification of Evidence Syntheses

The 8614 references were screened on title/abstract‐stage by one author (DT) using Covidence (https://www.covidence.org) according to the following inclusion criteria: 1. fully‐published, health‐related evidence syntheses including primary studies (not protocols or conference abstracts); 2. self‐describe as “systematic (literature) review,” “scoping (literature) review,” “meta‐analysis,” “comprehensive literature review,” “critical review” in title/abstract, or classified as publication type “Review” in the respective database and mention “systematic (literature) search” in the abstract; 3. mention “climate change,” “global warming,” or “climate‐health” in title/abstract. After the first screening, 348 references remained for inspection on the full‐text stage. These were re‐assessed for the inclusion criteria by another author (KJ, MIM, IM, LSW) and discrepancies resolved with a third author. We excluded further 139 evidence syntheses after assessment of the full‐text.

The remaining 209 evidence syntheses were extracted (bibliographic details, publication type) and classified per pathway by two independent authors (MIM + IM, KJ + LSW). Ten evidence syntheses were piloted with the team before conducting the full classification. If the evidence synthesis covered more than one pathway, we classified it into all applicable pathways and if it did not fit into a pathway, we classified it as “miscellaneous.” This latter category was re‐assessed and further classified by one author (DT) using free text words. In the last round, we further excluded 99 evidence syntheses according to the following criteria: 1. thematic focus was not related to an exposure pathway (e.g. policies, research methods, professional roles, education, economics related to climate change); 2. it lacked sufficient information about its included studies (e.g. no citation). This resulted in a final set of 110 relevant evidence syntheses remaining for study extraction (see Appendix 2).

#### 2.1.3 Extraction and Classification of Studies

Studies were extracted and classified by one member of the author team. However, during the classification process difficult cases were discussed within the team and approximately 25% were randomly checked by the first author (MIM).

We first extracted the number of included studies of the 110 identified relevant evidence syntheses. To ensure heterogeneity of topics, we then sorted them by their number of included studies (6 to 1920) and started extracting the evidence syntheses with the lowest number of studies. We skipped extraction of an evidence synthesis after reaching approximately 350 references per pathway. We extracted each included study (bibliographic data, study design) and classified it into one or several pathways. We also checked if the study was indexed in PubMed and recorded the identifier (PMID). If the study was not indexed in PubMed, we extracted the digital object identifier (DOI). After finishing, we had extracted 79 evidence syntheses published between 2008 and 2021, containing in total 2324 studies. During classification, we categorised 388 of these studies as not relevant, for which the reasons were: 1. not a primary study (but an evidence synthesis); 2. not related to climate change (e.g. earthquakes, volcanic eruptions, air pollution caused by traffic); 3. not adjudicable to a pathway. This resulted in a set of 1,928 relevant references, of which 1665 were indexed in PubMed. After removing duplicates, a final set of 1572 unique references of primary studies indexed in PubMed was available for establishing the development and validation sets (see Table 2).

**Table 2:** Reference set.

Pathways	ES extracted (*n*)	References extracted (*n*)	PMIDs with duplicates (*n*)	Relevant PMIDs without duplicates (*n*)	References indexed in PubMed (%)	Range of publication year of included studies
Air quality	25	360	339	286	94.2	1990–2019
Extreme weather events	27	298	244	229	81.9	1967–2022
Food supply and safety	17	167	135	98	80.8	1967–2018
Heat stress	33	439	401	339	91.3	1959–2021
Social factors	7	73	29	28	39.7	1990–2020
Vector distribution and ecology	28	419	341	322	81.4	1946–2019
Water quality and quantity	20	392	351	270	89.5	1972–2018
not relevant†	—	388	263	—	—	—
Total‡	79	2324§	1928	1572	86.0	—
of those relevant	—	1936	1665	—	—	—

Abbreviations: ES, evidence syntheses; PMIDs, PubMed identifier.

^†^ 1. not a primary study; 2. not related to climate change; 3. not adjudicable to a pathway.

^‡^ N of total ES/references (not sum of pathways, because classification into several pathways possible).

^§^ References with 2–5 pathways = 189 (175 with 2 pathways, 14 with > 2 pathways).

### 2.2 Developing Graded Search Filters Per Pathway

A sample size of at least 100 references is required as a validation set for search filters, if the desired sensitivity is 0.9 (to establish a 95% confidence interval of 0.84 to 0.96) [13]. Therefore, we aimed to identify at least 200 references per pathway.

After establishing the reference set, we split the references per pathway into a validation set (100 references) and a development set (the rest of available references per pathway). For this purpose, we inserted all unique identified PMIDs per pathway in a spreadsheet, randomised them using ‘random. org’ (https://www.random.org), used the first 100 PMIDs for validation purposes and the rest for development. This resulted in a development set of 186 PMIDs for “air quality,” 129 for “extreme weather events,” 239 for “heat stress,” 222 for “vector distribution and ecology,” and 170 for “water quality and quantity.” For two pathways we did not meet our objective of collecting at least 200 PMIDs. For “food supply and safety” we only collected 98 PMIDs and used 60% for development (58 PMIDs) and 40% for validation (40 PMIDs). For the pathway “social factors” we only collected 28 PMIDs. As we considered this insufficient, we decided against developing a filter for this pathway.

We then proceeded to import the PMIDs of each pathway´s “development set” 1. into Voyant Tools (https://voyant-tools.org) for analyzing phrases up to four words from the references´ titles and abstracts (tiab), and 2. into PubReMiner (https://hgserver2.amc.nl/cgi-bin/miner/miner2.cgi) for analysing single words from references´‐tiab and the Medical Subject Headings (MeSH) associated with the references. We exported the outputs of each tool (frequencies of single words, phrases, and MeSH terms) into a spreadsheet for visual analysis and search term selection (separate columns for tiab‐phrases, tiab‐single words, and MeSH terms).

Two authors (MIM, IM) inspected: 1. all tiab‐single terms that were included in at least five references and selected 25 potential terms for testing; 2. all tiab‐phrases of two, three, and four words that were included in at least three references and selected 25 potential phrases for testing; 3. all MeSH‐terms that were included in at least 20 references and selected 15 potential MeSH for testing. During selection, we chose the most frequent and at the same time most specific terms for each pathway.

We then proceeded to test all selected terms (single words, phrases, MeSH) per pathway for their performance in retrieving the references of the respective development set (sensitivity) and, in addition, recorded the number of hits they retrieved in PubMed. For each selected term, we then divided the number of PubMed hits retrieved through its sensitivity. This resulted in a proxy for the best performing terms, which we added incrementally to build a search string with the Boolean Operator “OR” until we had reached the pre‐defined cut‐offs of 95%, 97% and 99% sensitivity. We then proceeded to validate the search filters and calculated the sensitivity of all three search strings in their validation sets.

Two authors (MIM, IM) developed the filters for the PubMed search interface and translated them to Ovid syntax (available as Appendix 3).

## 3 Results

Based on a dataset with 1572 primary study references indexed in PubMed, it was possible to develop graded search filters with sensitivity cut‐offs of 95%, 97% and 99% for six of the seven exposure pathways. The filters and their performance indicators are presented in Table 3 (PubMed syntax) and Appendix 3 (Ovid syntax). We did not develop a search filter for the pathway ‘social factors’ because of its low coverage in PubMed.

**Table 3:** Search filters per exposure pathway with their respective performance indicators.

Sensitivity cut‐off	95%	97%	99%
**Air quality**			
S: development	96.2	97.8	99.5
S: validation	100	100	100
Hits	347,817	447,543	622,073
Filter	WILDFIRE*[tiab] OR PM10[tiab] OR “pm 10”[tiab] OR Fires[mh] OR PM2[tiab] OR “air pollution”[tiab] OR “air quality”[tiab] OR “pm 2.5”[tiab] OR OZONE[tiab] OR “Particulate Matter”[mh] OR WEATHER[tiab] OR Smoke[mh] OR POLLUTANT*[tiab] OR HUMID*[tiab]	WILDFIRE*[tiab] OR PM10[tiab] OR “pm 10”[tiab] OR Fires[mh] OR PM2[tiab] OR “air pollution”[tiab] OR “air quality”[tiab] OR “pm 2.5”[tiab] OR OZONE[tiab] OR “Particulate Matter”[mh] OR WEATHER[tiab] OR “Air Pollution”[mh] OR Smoke[mh] OR POLLUTANT*[tiab] OR HUMID*[tiab] OR AMBIENT[tiab]	WILDFIRE*[tiab] OR PM10[tiab] OR “pm 10”[tiab] OR Fires[mh] OR PM2[tiab] OR “air pollution”[tiab] OR “air quality”[tiab] OR “pm 2.5”[tiab] OR OZONE[tiab] OR “Particulate Matter”[mh] OR WEATHER[tiab] OR “Air Pollution”[mh] OR Smoke[mh] OR POLLUTANT*[tiab] OR HUMID*[tiab] OR AMBIENT[tiab] OR ASTHMA[tiab]
**Extreme weather events**			
S: development	96.9	97.7	100
S: validation	91.0	95.0	97.0
Hits	305,696	320,948	470,148
Filter	RIVER*[tiab] OR Floods[mh] OR “Cyclonic Storms”[mh] OR HURRICANE*[tiab] OR FLOOD*[tiab] OR DISASTER*[tiab] OR DROUGHT*[tiab] OR Disasters[mh] OR WEATHER[tiab]	RIVER*[tiab] OR Floods[mh] OR “Cyclonic Storms”[mh] OR HURRICANE*[tiab] OR FLOOD*[tiab] OR DISASTER*[tiab] OR DROUGHT*[tiab] OR Disasters[mh] OR WEATHER[tiab] OR RAINFALL*[tiab]	RIVER*[tiab] OR Floods[mh] OR “Cyclonic Storms”[mh] OR HURRICANE*[tiab] OR FLOOD*[tiab] OR DISASTER*[tiab] OR DROUGHT*[tiab] OR Disasters[mh] OR WEATHER[tiab] OR RAINFALL*[tiab] OR Climate[mh]
**Food supply and safety**			
S: development	96.6	98.3	100
S: validation	100	100	100
Hits	278,693	338,598	482,844
Filter	“Child Nutrition Disorders”[mh] OR SALMONELLOS*[tiab] OR STUNT*[tiab] OR Cholera[mh] OR DROUGHT*[tiab] OR UNDERWEIGHT[tiab] OR Rain[mh] OR “Food Supply”[mh] OR FOODBORNE[tiab] OR Droughts[mh] OR MALNUTRITION[tiab] OR “Salmonella Infections”[mh] OR “Foodborne Diseases”[mh] OR WEATHER[tiab]	“Child Nutrition Disorders”[mh] OR SALMONELLOS*[tiab] OR STUNT*[tiab] OR Cholera[mh] OR DROUGHT*[tiab] OR UNDERWEIGHT[tiab] OR Rain[mh] OR “Food Supply”[mh] OR FOODBORNE[tiab] OR Droughts[mh] OR MALNUTRITION[tiab] OR “Salmonella Infections”[mh] OR “Foodborne Diseases”[mh] OR WEATHER[tiab] OR SALMONELLA*[tiab]	“Child Nutrition Disorders”[mh] OR SALMONELLOS*[tiab] OR STUNT*[tiab] OR Cholera[mh] OR DROUGHT*[tiab] OR UNDERWEIGHT[tiab] OR Rain[mh] OR “Food Supply”[mh] OR FOODBORNE[tiab] OR Droughts[mh] OR MALNUTRITION[tiab] OR “Salmonella Infections”[mh] OR “Foodborne Diseases”[mh] OR WEATHER[tiab] OR “Food Microbiology”[mh] OR SALMONELLA*[tiab] OR Seasons[mh]
**Heat stress**			
S: development	96.7	97.9	99.2
S: validation	98.0	98.0	98.0
Hits	483,853	643,922	783,400
Filter	“Extreme Heat”[mh] OR “daily mortality”[tiab] OR “heat wave*“[tiab] OR “heat related”[tiab] OR “extreme heat”[tiab] OR HEATWAVE*[tiab] OR “Heat Stress Disorders”[mh] OR WEATHER[tiab] OR METEOROLOGIC*[tiab] OR “ambient temperature*“[tiab] OR “climate change”[tiab] OR “air pollution”[tiab] OR SUMMER*[tiab] OR “Hot Temperature”[mh] OR Seasons[mh]	“Extreme Heat”[mh] OR “daily mortality”[tiab] OR “heat wave*“[tiab] OR “heat related”[tiab] OR “extreme heat”[tiab] OR HEATWAVE*[tiab] OR “Heat Stress Disorders”[mh] OR WEATHER[tiab] OR METEOROLOGIC*[tiab] OR “ambient temperature*“[tiab] OR “climate change”[tiab] OR “air pollution”[tiab] OR SUMMER*[tiab] OR “Hot Temperature”[mh] OR CLIMATE*[tiab] OR Seasons[mh] OR HOT[tiab]	“Extreme Heat”[mh] OR “daily mortality”[tiab] OR “heat wave*“[tiab] OR “heat related”[tiab] OR “extreme heat”[tiab] OR HEATWAVE*[tiab] OR “Heat Stress Disorders”[mh] OR WEATHER[tiab] OR METEOROLOGIC*[tiab] OR “ambient temperature*“[tiab] OR “climate change”[tiab] OR “air pollution”[tiab] OR SUMMER*[tiab] OR “Hot Temperature”[mh] OR CLIMATE*[tiab] OR Seasons[mh] OR HOT[tiab] OR Climate[mh] OR “high temperature*“[tiab] OR AMBIENT[tiab]
**Vector distribution and ecology**			
S: development	96.8	97.3	99.1
S: validation	95.0	95.0	96.0
Hits	1,028,716	1,135,744	1,663,279
Filter	RAIN*[tiab] OR Rain[mh:noexp] OR Climate[mh:noexp] OR “Insect Vectors”[mh:noexp] OR MOSQUITO*[tiab] OR “relative humidity”[tiab] OR “Climate Change”[mh:noexp] OR CLIMAT*[tiab] OR BORNE[tiab] OR Seasons[mh:noexp] OR ENDEMIC*[tiab] OR transmission[sh:noexp] OR “Disease Outbreaks”[mh:noexp] OR FEVER[tiab]	RAIN*[tiab] OR Rain[mh:noexp] OR Climate[mh:noexp] OR “Insect Vectors”[mh:noexp] OR MOSQUITO*[tiab] OR “relative humidity”[tiab] OR “Climate Change”[mh:noexp] OR CLIMAT*[tiab] OR BORNE[tiab] OR Seasons[mh:noexp] OR ENDEMIC*[tiab] OR transmission[sh:noexp] OR “Disease Outbreaks”[mh:noexp] OR EPIDEMIC*[tiab] OR FEVER[tiab]	RAIN*[tiab] OR Rain[mh:noexp] OR Climate[mh:noexp] OR “Insect Vectors”[mh:noexp] OR MOSQUITO*[tiab] OR “Climate Change”[mh:noexp] OR CLIMAT*[tiab] OR HUMID*[tiab] OR BORNE[tiab] OR Seasons[mh:noexp] OR ENDEMIC*[tiab] OR transmission[sh:noexp] OR “Disease Outbreaks”[mh:noexp] OR EPIDEMIC*[tiab] OR FEVER[tiab] OR VECTOR*[tiab] OR SURVEILLAN*[tiab]
**Water quality and quantity**			
S: development	95.9	97.1	99.4
S: validation	94.0	95.0	99.0
Hits	360,427	465,591	695,171
Filter	“heavy rain*“[tiab] OR Cholera[mh] OR Floods[mh] OR “Vibrio cholerae”[mh] OR WATERBORNE[tiab] OR RAINFALL*[tiab] OR FLOOD*[tiab] OR DIARRHEAL[tiab] OR CHOLERA*[tiab] OR “Water Microbiology”[mh] OR “Water Supply”[mh] OR DIARRHOEA*[tiab] OR Climate[mh]	“heavy rain*“[tiab] OR Cholera[mh] OR Floods[mh] OR “Vibrio cholerae”[mh] OR WATERBORNE[tiab] OR RAINFALL*[tiab] OR FLOOD*[tiab] OR DIARRH*[tiab] OR CHOLERA*[tiab] OR “Water Microbiology”[mh] OR “Water Supply”[mh] OR Diarrhea[mh] OR Climate[mh]	“heavy rain*“[tiab] OR Cholera[mh] OR Floods[mh] OR “Vibrio cholerae”[mh] OR WATERBORNE[tiab] OR RAINFALL*[tiab] OR FLOOD*[tiab] OR DIARRH*[tiab] OR CHOLERA*[tiab] OR “Water Microbiology”[mh] OR “Water Supply”[mh] OR Disasters[mh] OR Climate[mh] OR SEASON*[tiab]

*Note:* S: sensitivity (definition: number of records in a set retrieved by the search filter divided by the total number of records in the set).

Hits: records retrieved by the filter in PubMed on September 09, 2025.

All search filters in PubMed syntax; Ovid MEDLINE syntax translation available in File S1.

The ‘hits in PubMed’ are the numbers of records retrieved by running the filters in PubMed on May 12, 2024. The filters yielded between 255,000 to 956,000 records in their 95% sensitivity versions and between 435,000 to 1,538,000 records in their 99% sensitivity versions. This indicator can help researchers to choose a filter version in addition to considering its sensitivity. Of course, the number of retrieved records depends on the prevalence of the pathway in PubMed. In practice, the filters are intended to be combined with other concepts (e.g. geographic region, specific populations) which will reduce the number of retrieved records.

The indexing of the studies in PubMed showed a marked difference between the pathways, with three clusters emerging. Studies for “air quality,” “heat stress,” and “water quality and quantity” had a high indexing rate between 90% and 94%, while “extreme weather events,” “food supply and safety,” and “vector distribution and ecology” had an indexing rate of 81%–82%. The pathway “social factors” had an exceptionally low indexing rate of 40% (Table 2). This observation could be related to the respective topic (pathway) coverage in PubMed itself and the different time points when topics started being investigated, which is reflected in the range of publication years of the included studies per pathway (Table 2).

## 4 Discussion

Our aim was to develop robust and graded search filters for major exposure pathways linking climate change and health, which are essential for identifying relevant literature and synthesizing this expanding body of evidence on climate change and health. To our knowledge, this manuscript contributes the first search filters for pathways linking climate and health. While it was possible to develop filters for five pathways as planned, we only gathered half the intended number of references for “food supply and safety” and are therefore less confident that its sensitivity is close to its true performance. This should be considered when using this pathway´s filters and we recommend using the 97% or 99% versions.

The number of records retrieved with our filters per exposure pathway might seem high. However, we would like to emphasize that the number of results is a reflection of the pathways´ scope. We assume that during deployment the filters will be combined with further concepts according to the research question of interest, which will limit the total number of hits retrieved. For example, the “air quality”‐filter can be combined with a specific extreme weather event (“storms”) to identify literature investigating impacts of particulate resuspension after a storm: [99% “air quality”‐filter] AND (storm[tiab] OR storms[tiab] OR thunderstorm*[tiab] or windstorm*[tiab]), retrieved 3278 hits on July 25, 2026, showing how these filters can be used in practice.

For the pathway “social factors,” we desisted from filter development because of the limited number of PubMed references identified, suggesting that identifying studies related to this pathway will likely require searching additional databases and grey literature, or using citation searching. To be able to develop a robust search filter for this specific pathway in the future, we would need to revisit systematic reviews evaluating the impacts of climate change on social factors in a few years, to analyze if the relevant studies included in these reviews are still mostly indexed outside of PubMed or if this has changed because the topic is increasingly being investigated and covered by journals indexed in PubMed.

The main strengths of our filters are that they will help researchers to identify relevant primary studies that do not mention their relationship to climate change, they have been empirically developed by extracting and analyzing the existing literature, and they can be applied to different research questions (interventions, associations, impacts, diseases, populations, communities, or regions) by focusing on the major exposure pathways linking climate change and health.

Some limitations must be acknowledged. Firstly, the reliance on a single person for ¾ of the data extraction and classification may have introduced errors. While difficult cases were discussed in the team, the possibility of misclassifications or overlooked pathways cannot be discarded. Additionally, we acknowledge our limited expertise in some of the pathway domains, which could also have resulted in classification errors. Another limitation arises from the fact that the sample size calculation we used as basis for our filters was orginally suggested for methodological filters [13]. Our search filters cover exposures, which potentially implies a wider variation in terminology and we acknowledge that the empirical derivation of terms was limited by the sample size. The optimum sample size for subject filters is not known and can be expected to differ per topic, therefore calculation efforts have not been attempted. This needs to be taken into account with regard to the performance measures of our filters. In addition, the relative recall method relies on the quality of the searches underpinning the evidence syntheses used for extraction of primary studies. While we did not assess the quality of the evidence syntheses nor their searches, we only included reviews that claimed to be systematic or have used a systematic approach to searching. Last but not least, it would be beneficial to have an independent evaluation of our filters to additionally help assess their performance. We also expect that climate‐health language will change in the future. As terminology evolves and new concepts emerge, it will be essential to re‐validate the search filters. Applying the filters in other databases requires translating their syntax, including the mapping of MeSH terms to the controlled vocabulary used in those databases. However, this does not guarantee equivalent performance, and further validation is necessary to confirm their effectiveness in other databases.

As Minx and colleagues argue, anthropogenic climate change is a wicked problem for which evidence‐based policy responses are urgently needed [16]. Evidence syntheses presenting actionable messages for policymakers will enable initiatives to avoid, prepare for, or manage the risks of climate change. Appropriate methods for such syntheses are needed and the health community needs to learn from other disciplines and promote intersectoral collaboration [16, 17]. Thus, we recommend that health researchers conducting climate‐health evidence syntheses consider to: 1. use validated search filters to help capture relevant primary studies with high sensitivity and collaborate with medical librarians when developing literature search strategies; and 2. prepare to incorporate sources of evidence not traditionally used in health‐related evidence syntheses, such as non‐randomised studies, qualitative data, and modelling studies in situations where direct evidence is not available [9]. Further research regarding information retrieval methods could include the development of search filters in different languages, explore the possibility of constructing filters for the pathway “social factors” by examining the indexing in databases such as Web of Science or CABI Global Health, and independently validate and update the filters as climate‐health terminology evolves and the evidence base expands.

## 5 Conclusions

We developed and validated search filters with a graded sensitivity for six of seven major exposure pathways linking climate change and health for the MEDLINE database and presented them for two main search interfaces (PubMed and Ovid). They can be readily used by health information professionals and researchers conducting primary research and evidence syntheses on climate‐health who want to overcome some of the described challenges associated with the identification of the evidence base on climate change and health. The availability of these filters represents a critical step towards helping to synthesise the growing body of evidence on the links between health and climate change and inform policy‐making and public health interventions addressing the impacts and mitigation of and adaptation to the developing climate emergency.

## Author Contributions

**Maria‐Inti Metzendorf:** writing – original draft, conceptualization, methodology, validation, visualization, formal analysis, data curation, investigation. **Ina Monsef:** writing – review and editing, validation, methodology, formal analysis, data curation, investigation. **Katherine Jones:** data curation, writing – review and editing, methodology. **L. Susan Wieland:** writing – review and editing, methodology, data curation. **Heidrun Janka:** writing – review and editing, data curation. **Camila Escobar Liquitay:** writing – review and editing, data curation. **Denise Thomson:** methodology, data curation, writing – review and editing.

## Ethics Statement

This study did not require ethics approval as it is a methodological study utilising published data. The data analysed were obtained from published evidence syntheses, ensuring compliance with ethical standards for research involving secondary data analysis.

## Consent

The authors have nothing to report.

## Conflicts of Interest

The authors declare no conflicts of interest.

## Reporting Checklist

A reporting checklist for search filters is currently not available. However, we consulted the “critical appraisal of search filters form” established by the UK‐based InterTASC Information Specialists' Sub‐Group, which is published on their Search Filter Resource (https://sites.google.com/a/york.ac.uk/issg-search-filters-resource/home/critical-appraisalof-search-filters) and have checked that we report on all the main items covered in this form.

## Supporting information


Supporting File

## Data Availability

All data relevant to the study are included in the article or the supporting information. The full data sets of extracted studies and full details of the search filter development are available from the corresponding author upon reasonable request.
